# Diabetes NetPLAY: A physical activity website and linked email counselling randomized intervention for individuals with type 2 diabetes

**DOI:** 10.1186/1479-5868-6-18

**Published:** 2009-03-27

**Authors:** Tanis Liebreich, Ronald C Plotnikoff, Kerry S Courneya, Normand Boulé

**Affiliations:** 1 Centre for Health Promotion Studies, School of Public Health, 5-10 University Terrace, 8303 - 112 Street, University of Alberta, Edmonton, Alberta, T6G 2T4, Canada; 2 Centre for Health Promotion Studies, School of Public Health, and Faculty of Physical Education and Recreation, 5-10 University Terrace, 8303 - 112 Street, University of Alberta, Edmonton, Alberta, T6G 2T4, Canada; 3 Faculty of Physical Education and Recreation, E-488 Van Vliet Centre, University of Alberta, Edmonton, Alberta, T6G 2H9, Canada

## Abstract

**Background -:**

This pilot study evaluated the feasibility (*recruitment, retention, adherence and satisfaction*) and preliminary efficacy of a 12-week website and email-linked counselling intervention on physical activity behaviour change in individuals with type 2 diabetes.

**Methods -:**

A total of 49 individuals with type 2 diabetes (59% female, average age 54.1 years) were randomized to the Diabetes NetPLAY intervention or control condition. The intervention condition received information grounded in the Social Cognitive Theory (SCT), personalized weekly emails, an on-line logbook and message board. Key outcomes included physical activity behaviour and related cognition changes. The control condition was provided links to the Canadian Diabetes Association's Clinical Practice Guidelines for Physical Activity and Canada's Guide to Physical Activity.

**Results -:**

Intervention participants indicated high levels of satisfaction for this mode of delivery and study results demonstrated the feasibility of web-based mediums for the delivery of physical activity information in this population. The intervention group demonstrated a significant improvement in total vigorous and moderate minutes of physical activity (p = 0.05) compared to the control group over the 12-week study. Among the SCT variables, behavioural capacity, showed a significant increase (p < 0.001) among intervention participants.

**Conclusion -:**

Web-based interventions for individuals with type 2 diabetes are feasible and show promise for improving positive physical activity outcomes.

## Background

Physical activity has long been recognized as one of the cornerstones of diabetes management [1]. Physical activity, including aerobic and resistance training, can assist individuals with type 2 diabetes in achieving a variety of goals including improved glycemic control, increased cardiorespiratory fitness, decreased insulin resistance, improved lipid profile and weight management [2]. Moderate to high levels of cardiorespiratory fitness in those with type 2 diabetes has been associated with a 45–70% reduction in both cardiovascular and overall mortality [3].

The Canadian Diabetes Association's Clinical Practice Guidelines recommend those with type 2 diabetes accumulate at least 150 minutes of moderate intensity aerobic activity per week spread over at least three non-consecutive days [4]. However, individuals with diabetes report high inactivity rates, with more than 60% of adults not meeting recommended physical activity guidelines [5,6].

The internet offers a unique opportunity for delivering innovative, large scale behavioural change interventions, including physical activity [7-10]. Several advantages exist for internet health technology. Information is available to users 24 hours a day allowing for information to be viewed at their convenience. It also houses components such as chat rooms and web conferencing which can facilitate social support and communication from other users and health care providers [11]. However, the internet can pose obstacles for individuals as well. Low-income households report reduced rates of computer and internet usage [12]. Access to internet technology in rural areas can be limited or only available via dial-up technology. Literacy levels and computer skills are also potential barriers that individuals may face when using this type of technology to access health information.

According to Statistics Canada [12], 64% of Canadian households had at least one member who accessed the Internet regularly either from home, work, school, public library or another location. Of internet users, 84% report having access to email with 39% using it everyday to communicate and 25% making use of email at least once a week. Additionally, more people are using the internet to find health information, making it the third most searched topic behind email and general browsing. For these reasons the internet, and specifically email, can be considered as a potential channel for the administration and delivery of physical activity interventions [13].

The use of interactive technology to change behaviour is expanding the scope and flexibility of intervention and teaching options [14]. The use of internet technology and e-counselling techniques has shown promising results in the weight loss [15,16], nutrition [17,18], and physical activity domains [13,19-23].

In the physical activity domain, Plotnikoff and colleagues [19] assessed the efficacy of a 12-week email physical activity and nutrition intervention on knowledge, attitude and behaviour change in a large workplace sample. At the conclusion of the study, the intervention group had significantly increased their total activity levels and reported higher confidence and intention levels related to physical activity participation compared to the control group.

Napolitano et al. [13] tested the efficacy of a theoretically-based physical activity website plus 12 weekly email tip sheets on a sample of sedentary employees of several large hospitals. At 1-month follow-up, participants in the intervention group demonstrated significantly more minutes of moderate physical activity and walking minutes per week. Additionally, a greater proportion of those in the intervention group progressed in stage of motivational readiness than those in the control. At 3-months follow-up, those individuals in the intervention group were more likely to progress in stage of motivational readiness than individuals in the control.

There is a growing number of websites targeting diabetes education and support; however, the use of interactive computer technology to increase physical activity behaviour in individuals with type 2 diabetes is in its infancy. In light of this new technology base there is little empirical evidence that such interventions improve the outcomes and quality of life of those who participate in them [8,24].

In one of the first studies in this domain, McKay and colleagues [11] conducted an 8-week pilot study to evaluate the feasibility and impact of the Internet-based Diabetes Network (D-Net) Active Lives Physical Activity Intervention. A total of 78 individuals with type 2 diabetes were randomized to the D-Net intervention site or to an internet-information only condition. At the 8-week follow-up, results indicated an overall moderate improvement in physical activity levels within both groups, with no significant improvement in regard to condition effects. Among participants in the intervention group, those who utilized the site more regularly obtained significantly greater benefits compared to the control group who demonstrated only modest improvements with increased program use.

Theoretical approaches have been used not only to understand physical activity as a behaviour but also to provide the conceptual and empirical knowledge base for the design of activity promoting programs. To provide guidance for the design of effective programs, such as web-based strategies, interventions must be grounded in behaviour change theory [25].

One of the major behaviour change theories is the Social Cognitive Theory (SCT) as presented by Bandura [26], which postulates that personal, behavioural, and environmental factors operate as reciprocal interacting determinants of human functioning. Furthermore, the notion of reciprocal determinism suggests that individuals are both agents and recipients of their behaviours. According to Bandura [27,28] a number of crucial factors influence behaviour. These core determinants include knowledge of health risks and benefits of different health practices, perceived self-efficacy that one can exercise control over their individual health habits, the health-related goals they set for themselves and the specific plans and strategies for realizing them, as well as the perceived facilitators and impediments to the changes they strive for. Other critical factors included in the SCT are the individuals' capabilities to symbolize behaviour, to learn by observing others, to have confidence in performing a behaviour, to self-regulate or self-determine behaviour and to reflect on and analyze experience [27]. Baranowski, Perry & Parcel [29] present the social cognitive theory as ten specific constructs: self-efficacy, self-regulation, outcome expectations, outcome expectancies, behavioural capacity, observational learning, reinforcement, emotional coping response, perceived environment and situation.

SCT has been acknowledged as one of the leading health behaviour change theories used to explain and predict physical activity [30,31] in the general population and in those with type 2 diabetes [32]. SCT has been applied to several diabetes management studies aimed at increasing physical activity in this population [33,34]. The First Step Pedometer Program developed by Tudor-Locke and colleagues [33] operationalized self-efficacy and outcome expectations from SCT with the short-term goal of increasing physical activity (walking) in overweight/obese individuals with diabetes. At the conclusion of the 24-week follow-up, the intervention group had increased their physical activity by 3000 steps/day or approximately 30 minutes/day (p < 0.001) compared with that of the control group.

Di Loreto and colleagues [34] developed a counselling strategy, based on self-efficacy and outcome expectations constructs from SCT, to increase physical activity behaviour in individuals with type 2 diabetes. In this study, 342 individuals were randomized to either an intervention or control group. At the two-year follow-up, results indicated the intervention group, compared to the control group, significantly increased energy expenditure seven-fold (p < 0.001) with significant improvements also reported in HbA1c and BMI.

In summary, the use of web-based technology to deliver physical activity information relevant to those living with type 2 diabetes is expanding. However, further research on designing and testing theoretically grounded web-based interventions is needed for changing health behaviour in this population.

The purpose of this study is to explore the feasibility and preliminary efficacy of a website and email-linked counselling intervention on physical activity behaviour change in individuals with type 2 diabetes. We examine the feasibility (*recruitment*, *retention*, *adherence *and *satisfaction*) of the internet as a mode of delivery for diabetes related physical activity information for individuals with type 2 diabetes. In addition, the *preliminary efficacy *of the internet as a mode of delivery for eliciting recommended changes in physical activity related cognitions and behaviours for individuals with type 2 diabetes is also examined. We hypothesize that physical activity-related cognitions and behaviours will increase in the intervention group when compared to the control group after 12 weeks.

## Methods

### Design

The study was a prospective, two-armed, randomized controlled trial. Participants were randomly assigned to either the intervention or control condition.

### Recruitment

A sample of men and women with type 2 diabetes were recruited through diabetes education classes, newspaper advertisements, recruitment posters, health care professionals and previous research involvement. Interested respondents were screened via telephone/in person for participation criteria and, if eligible, informed consent was obtained. Inclusion criteria included a diagnosis of type 2 diabetes, being over the age of 18 and having access to the internet and email. Contraindications for physical activity were accessed by the Physical Activity Readiness Questionnaire (PAR-Q) [35]. Participants who indicated a contraindication to physical activity based on the PAR-Q were required to provide written consent from a primary care physician.

**Figure 1:**
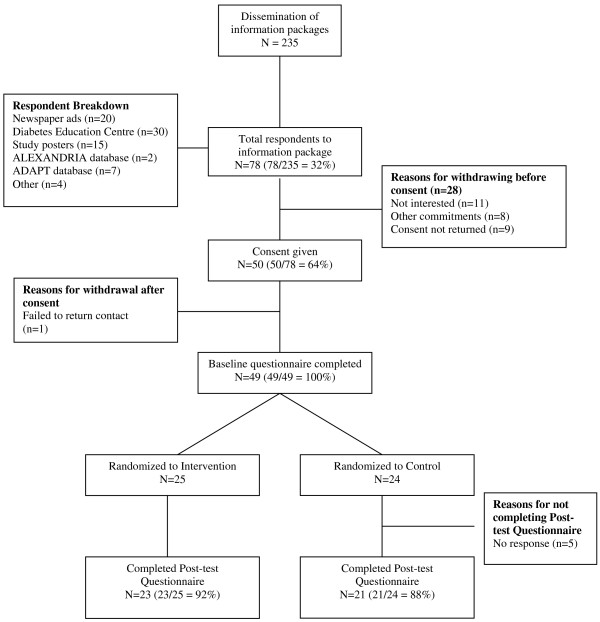
**Study Flow Diagram**.

A total of 235 study information packages were distributed over the 3 month recruitment period. Seventy-eight individuals responded to the information packages, with 50 providing consent to participate. Of those individuals that did not give consent, 11 were not interested in the study, 8 indicated other commitments and another 9 failed to return the required consent forms. One individual failed to be reached after consent to participate in the study was given (see Figure 1: Study Flow Diagram). Ethical clearance for this study was obtained from a university-based, ethics committee.

### Procedures

Upon recruitment deadline, all eligible participants completed an on-line questionnaire assessing baseline measurements of physical activity behaviour, physical activity-related cognitions and demographic characteristics. Upon completion, participant names were randomized with equal probability into one of two groups: intervention group or the control group. A total of 49 individuals with type 2 diabetes were randomly assigned into the control condition (n = 24) or Diabetes NetPLAY intervention group (n = 25). Participants were then informed via email of their group assignment and were provided with confidential usernames and password to access the appropriate website.

### Diabetes NetPLAY intervention group

The Diabetes NetPLAY program was designed based on Bandura's [27] Social Cognitive Theory (SCT) and aimed to provide participants with the education and skills important for long term behaviour change. The website was comprised of five main sections (weekly topic, education, research, fitness tips and physical activity myths) which were updated and archived on a weekly basis. The website also contained several interactive features to engage participants through the duration of the study. These features included a physical activity logbook, message board and weekly email counselling from the study coordinator. Participants in the intervention group also received links to the Canadian Diabetes Association's Clinical Practice Guidelines [4] for physical activity in addition to Health Canada's Physical Activity Guide .

Each week featured a new theme based on a specific construct of SCT. This weekly topic aimed to operationalize the SCT construct in a way that would be meaningful to the participant. As a way of invoking thoughts and actions around the various constructs, participants were asked to complete an activity and email responses to the study coordinator. Goal setting and time management were some of the activities discussed.

#### Physical activity logbook

Participants in the intervention group were encouraged to log their physical activity minutes using the on-line logbook section of the website. The logbook allowed for participants to track the date, length, and type of physical activity they engaged in. This feature also allowed them to write comments beside their activity sessions and track their progress from day-to-day.

#### Message board

The message board feature was launched in the second week of the study. The study coordinator posted weekly topics based on the social cognitive construct featured that week. Participants could respond and view postings at their leisure. This allowed participants a forum in which to exchange ideas and receive support from other study participants.

#### Email counselling

The email counselling feature of the website allowed participants the opportunity to communicate with their counsellor (study coordinator) in a simple and efficient way. Beginning in the first week, participants were asked to email their counsellor with responses to the relevant topic discussed; however, participants could communicate with their counsellor via email at any time throughout the week on topics of importance to them. If participants failed to contact their counsellor on any given week, a brief email checking in on the participant was sent as a reminder.

Qualifications for the study counsellor included a Bachelors of Science in Physical Education and a Masters of Science in Health Promotion with a strong focus in behaviour change theory, diabetes and physical activity.

### Control ('Standard Care') condition

The control group received static links to the Canadian Diabetes Association's Clinical Practice Guidelines [4] for physical activity and Health Canada's Physical Activity Guide (as 'Standard Care' material) on the control website for the duration of the 12-week study. Control participants were not given any specific instruction/indication on what their physical activity behaviour should entail for the proceeding 3 months (study commencement). Participants were contacted at the end of week 12 and provided a link to the post-test questionnaire. Upon completion of the post-test questionnaire, control group participants were given full access (minus email counselling feature) to the study website. As the website was no longer being maintained and updated, these participants were directed to the archived items to review each of the 12 weeks at their leisure. Incentives were offered to control participants upon completion of the post-test questionnaire. Incentives included full access to the study website and a choice of a pedometer and water bottle or a $25 gift certificate to a national bookstore chain.

### Measures

All measures were administered on-line at baseline and at the end of the 12-week study. Reminder emails were sent if participants failed to complete questionnaires within 10 days of initial administration.

Baseline demographic variables were included based on various socio-demographic measures from Statistics Canada [36]. Participants were asked age, gender, marital status, English as a first language, education, current employment status, and gross annual family income. A physical activity disability measure was assessed using previously published self-report measure [6]. Participants were also asked to report their height (in feet/inches or meters/centimetres) and weight (in pounds or kilograms), in order to calculate BMI (kg/m^2^). BMI categories were defined by Health Canada [37]. Finally, computer usage data were collected using items from Statistics Canada's Household Internet Use Survey [12].

#### Physical Activity

Leisure-time physical activity (LTPA) was assessed using a modified version [6] of the validated Godin Leisure-Time Exercise Questionnaire (GLTEQ) [38]. In this instance, LTPA was defined as a physical activity session longer than 10 minutes in duration and was not part of employment or household chores. Based on an average of the past month, participants were asked to report: (1) frequency (times per week), and, (2) duration (average time per session), of activity in each intensity category (strenuous, moderate or mild). The reliability of the GLTEQ has been independently evaluated and found to compare favourably to nine other measures of self-report exercise, objective monitors, and fitness indices [39]. Participation responses were then converted into: (1) MET minutes by multiplying weekly minutes of moderate activity by 4 METS and vigorous activity by 7.5 METS [40]; and, (2) *unweighted *minutes by summing weekly minutes of moderate and vigorous physical activity.

Mild activity levels were not included in either calculation for three reasons: (1) all psychosocial variables are based on a definition of physical activity of moderate or greater intensity; (2) population-based MET values have only been defined for moderate activity or greater [40]; and, (3) the Canadian Diabetes Association's Clinical Practice Guidelines [4] state that moderate to high levels of physical activity are associated with substantial decreases in mortality and morbidity in individuals with type 2 diabetes. Although not included in the outcome measure, mild intensity physical activity is still important to the overall health of those individuals with type 2 diabetes, especially for those who have been sedentary in the past.

A resistance training (RT) measure was also incorporated into the GLTEQ in which participants were asked to report on average in the past month, frequency (times per week), and duration (average times per session) they had engaged in resistance training activities. Participation responses were captured by multiplying frequency by duration to produce a final RT score.

#### Social Cognitive Measures

##### Self-efficacy

Self-efficacy was assessed using a validated 12-item scale [41]. Each item was measured on a five-point Likert-type scale (1 = strongly disagree; 5 = strongly agree) and asked individuals to rate their confidence over the next 3 months in performing regular physical activity in a variety of circumstances (e.g., when tired, when busy, during bad weather).

##### Outcome expectations

Outcome expectations was assessed using a five-point Likert-type scale with response options ranging from "strongly disagree" (1) to "strongly agree" (5), and had participants rate their agreement with 17 expectation items of engaging in regular physical activity. A sample item from this scale was "I will feel better physically if I get regular physical activity." The outcome expectations measure was adapted from two sources: (1) the decisional balance scale originally designed by Marcus, Rawkoski, & Rossi [42], and adapted for a Canadian population by Plotnikoff et al. [43]; and (2) the physical activity expectations scale used in the PARR project [44]. Both sets if items were framed 'for the next 3 months.'

##### Outcome expectancies

The outcome expectancies scale had participants rate on a three-point Likert-type scale (1 = unimportant; 3 = very important) the perceived importance of the 17 previously stated expectation items. An example of an item from this scale was "How important is feeling better physically to you?" This scale was adapted from the PARR project [44], which had originally been created, tested and utilized with a low income population.

##### Self-regulation

The self-regulation measure was adapted from a subscale of the Behaviour Regulation in Exercise Questionnaire (BREQ-2) [45] identified for exercise regulation. The three-item scale had participants indicate how true a variety of reasons were (e.g., 'I value the benefits of exercise') for them participating in regular physical activity, with response items ranging from "not at all true" (1) to "very true" (5).

##### Situation

The situation construct was measured by having participants indicate how often in the past three months various situations prevented them from getting regular physical activity. Response options for this 17-item scale ranged from "never" (1) to "very often" (5) and were adapted from a previous physical activity scale used in a study of breast cancer survivors [46].

##### Reinforcement

Reinforcement was measured using a five-point Likert-type scale with responses ranging from "never" (1) to "always" (5). Participants were asked to specify how often in the last three months they had rewarded themselves and set realistic goals. A sample question from this four item scale developed by Marcus, Rossi, Shelby, Niaura and Abrams [47], is "I reward myself when I am physically active."

##### Social Support

The social support measure, previously used by Courneya, Plotnikoff, Hotz & Birkett [48], consisted of two items measured on a 7-point Likert-type scale with options ranging from "strongly disagree" (1) to "strongly agree" (7). A sample item of this scale was "Over the next three months, people in my social network are likely to help me participate in regular physical activity."

##### Emotional Coping Response

The emotional coping response measurement was adapted from the emotional well-being subscale [49] and assessed how participants felt about their diabetes over the past month. Response options for the five-item scale ranged from "not at all" (1) to "very much" (5). An example from this scale was: "I am proud of how I'm coping with my diabetes."

##### Behavioural Capacity

Behavioural capacity was measured using four items on a 5-point Likert-type scale in which participants were asked to rate how confident they were in performing specific tasks within the last three months. Response options ranged from "never" (1) to "always" (5). A sample item for this scale developed for patients with chronic disease [50] was: "I can walk briskly for 20 minutes without stopping."

##### Environment

The environment measure was assessed using an adapted form of the International Physical Activity Prevalence Study Environmental Survey Module [51]. Seven items asked participants to rate on a 5-point Likert-type scale, with options ranging from "strongly disagree" (1) to "strongly agree" (5), how given statements described the area in which they lived. For example, "There are sidewalks on most of the streets in my local area."

##### Observational Learning

Observational learning was measured using two items previously used by Plotnikoff et al. [6] on a 5-point Likert-type scale in which participants were asked to rate how often they observed others being active in the last three months. Response options ranged from "never" (1) to "very often" (5), with an example item being, "I have observed people who are important to me engaging in regular physical activity."

##### Satisfaction

Participants randomized to the intervention group completed a 15-item satisfaction survey adapted from the Health *e*-steps Program [52]. The survey was completed immediately post intervention and asked participants to rate on a Likert-type scale the satisfaction/usability of the intervention website. Items included the credibility and content of the information on the website as well as satisfaction of various components of the website with response options ranging from "strongly disagree" (1) to "strongly agree" (5). Three open-ended questions were asked to assess the likes and dislikes and recommended changes to the website.

For all above measures, 'regular physical activity' was defined as doing at least 150 minutes of moderate intensity physical activity at the pace of brisk walking or faster (to include vigorous activity) each week.

### Data Analysis

All analysis was conducted using SPSS (Version 12.0). Descriptive analysis (percentages and frequency counts) were conducted to assess retention, recruitment, adherence and satisfaction of the internet as a mode of delivery for physical activity information in the population.

Chi-square tests for independence were performed to examine group differences on pre-test categorical demographic scores. When applicable, demographic, health and computer usage variables were dichotomized into a 2 × 2 table to ensure the minimum expected cell frequency was not violated.

Repeated measures analysis of covariance (RM ANCOVA) was conducted to compare the efficacy of the NetPLAY intervention to increase physical activity levels and all 11 physical activity-related cognitions. The dependent variables for the primary outcome consisted of scores calculated on the modified Godin Leisure-Time Exercise Questionnaire (GLTEQ) to assess: (1) MET.minutes; and (2) unweighted, combined moderate and vigorous minutes of physical activity. Participant baseline physical activity scores were used as a covariate in the above analyses. We employed intention to treat (last observation carried forward) for missing values at post-test. An examination of the diabetes literature suggests an association between BMI and physical activity behaviour in those with type 2 diabetes [6,53]. For example, Plotnikoff and colleagues [6] surveyed a sample of 1600 individuals with type 2 diabetes and found higher activity levels were associated with lower BMI (p < 0.001), while Morrato and colleagues [53] found that for those with diabetes the probability of being active incrementally declined with each increasing BMI category. For this reason, along with the significant differences between the two study groups at baseline, BMI was included as a covariate (with baseline physical activity) in subsequent analysis for the primary behaviour outcome (i.e., physical activity).

## Results

### Baseline characteristics

Table 1 and Table 2 present the baseline data collected from a total of 49 participants (24 control, 25 intervention). The study groups did not statistically differ on any of the baseline characteristics measured with the exception of BMI (p < 0.05) and outcome expectations (p < 0.05).

**Table 1:** Baseline socio-demographic and behavioural characteristics of study participants

**Variable**	**Control (N = 24)** **Baseline** **M (SD)**	**Intervention (N = 25)** **Baseline** **M (SD)**	**t**	**p**
Current Age	54.5 (10.8)	53.7 (9.8)	-0.3	0.805
				
Age when diagnosed	47.5 (11.0)	46.5 (10.0)	-0.3	0.746
				
Time (in months) living with diabetes	86.6 (90.8)	86.9 (100.8)	0.0	0.992
				
BMI	31.1 (5.6)	36.6 (9.1)	2.5	**0.016**
				
MET Minutes (per/wk)	501 (582.0)	483 (620.0)	-0.1	0.987
				
Unweighted Minutes (per/wk)	111 (123.0)	105 (140.0)	-0.2	0.769
				
RT minutes (per/wk)	8 (23.0)	32 (62.0)	1.3	0.080
				
Disability	2.2 (1.5)	2.8 (1.9)	1.1	0.261
				
	**n (%)**	**n (%)**	χ^2^	
		
Marital Status				
Partner	20 (83.3%)	13 (52.0%)	4.14	**0.04**
No Partner	4 (16.7%)	12 (48.0%)		
				
Residence				
City	20 (83.3%)	16 (64.0%)	1.46	0.23
Not city	4 (16.7%)	9 (36.0%)		
				
Education				
No degree completed	5 (21.7%)	9 (36.0%)	0.59	0.44
Degree completed	18 (78.3%)	16 (36.0%)		
				
Gross Family Income				
< $20,000–$59,000	7 (30.4%)	12 (48.0%)	0.90	0.34
$60,000 – < $100,000	16 (69.6%)	13 (52.0%)		
				
Employment status				
Not employed	7 (29.2%)	9 (37.5%)	0.09	0.76
Employed	17 (70.8%)	15 (62.5%)		
				
Computer usage				
Less than once/week	10 (66.7%)	5 (33.3%)	0.60	0.44
More than once/week	9 (47.4%)	10 (52.6%)		
				
Computer usage				
Less than 20 hrs/month	18 (64.3%)	10 (35.7%)	4.78	**0.03**
More than 20 hrs/month	6 (28.6%)	15 (71.4%)		

**Table 2:** Baseline social cognitive variables – between groups

**Social Cognitive Variable**	α (or r*) **(# of items)**	**Control n = 24** **Baseline** **M (SD)**	**Intervention n = 25** **Baseline** **M (SD)**	**t**	**p**
self-efficacy	0.94 (12)	3.0 (0.7)	3.0 (0.7)	-0.1	0.947
outcome expectations	0.97 (17)	4.6 (0.3)	4.5 (0.9)	-0.8	0.041
outcome expectancies	0.88 (17)	2.7 (0.3)	2.7 (0.3)	0.2	0.810
self regulation	0.74 (3)	3.9 (0.5)	3.8 (0.5)	-0.9	0.392
situation	0.88 (17)	2.1 (0.5)	2.4 (0.7)	1.7	0.120
reinforcement	0.78 (4)	2.5 (0.8)	2.7 (0.7)	0.7	0.501
social support	0.83 (2)*	4.5 (1.9)	5.1 (1.5)	1.0	0.350
emotional coping resp.	0.71 (6)	2.7 (0.6)	2.9 (0.8)	0.8	0.450
behavioural capacity	0.85 (4)	3.2 (0.9)	3.1 (1.2)	-0.5	0.632
environment	0.77 (7)	3.6 (0.9)	3.5 (0.9)	-0.4	0.699
observational learning	0.29 (2)*	3.8 (1.3)	4.1 (0.8)	1.0	0.331

### Study Objective One: Study feasibility

The first objective of Diabetes NetPLAY was to determine the feasibility of the internet as a mode of delivery for diabetes related physical activity information for individuals with type 2 diabetes, specifically examining *recruitment*, *retention*, *adherence *and *satisfaction*. Regarding *recruitment*, approximately 185 individuals were mailed study information packages and another 50 packages were directly given to individuals attending diabetes education classes at a local hospital. Of the 235 packages distributed, 78 individuals requested further information about the study, representing a response rate of 33%. A total of 49 individuals consented to participate in the study, resulting in an overall recruitment rate of 21% (see Figure 1).

In terms of *retention*, two out of 25 participants from the intervention group did not complete the post-test questionnaire, representing an attrition rate of 8%. A total of three participants from the control group did not respond to the post-test questionnaire, signifying an attrition rate of 12.5% for this group (see Figure 1).

*Adherence *data showed that of the 25 participants randomized to the intervention group, 15 (60%) accessed the website at least once per week for a minimum of eight weeks (i.e., per protocol criteria). Every participant in the intervention group logged onto the website at least once throughout the study. Intervention group hits to the website ranged from one to 121 with a median of 22 hits per person during the course of the study. Login frequency decreased in 60% (n = 15) of the sample by study midpoint, 8% (n = 2) stayed the same while 32% of participants actually increased their frequency of website access. Of those participants who decreased their visits to the website in the latter six weeks of the study, only four individuals dropped off their usage by more than half. Email counselling participation varied among participants in the intervention group, with one respondent initiating contact over 10 times while another recorded no contact with the counsellor. One hundred and ten messages were received by the study counsellor over the 12 week intervention, representing an average of 4.4 messages per participant. Subsequent analysis did not reveal any correlation between website adherence and physical activity among intervention participants.

The median satisfaction score was 4.19 with a range of 3.41 to 4.50 out of five. Eighty-six percent of individuals either "strongly agreed" or "agreed" that the website was user-friendly while 95.5% found the information on the website to be easy to understand and credible. The various components of the website had more variability in satisfaction with 68.2% of participants reporting they either "agreed" or "strongly agreed" that the weekly activities were useful, and 77.3% stated the same for their satisfaction of the email counselling component. A detailed account of the satisfaction scores are presented in Table 3.

**Table 3:** Satisfaction with intervention program

**Variables**	**Intervention Group N = 22** **Mean (SD)**
Website was user friendly	4.27 (0.70)
Overall presentation	4.14 (0.71)
Able to find way around	4.23 (0.75)
Information was easy to understand	4.45 (0.60)
Information was credible	4.50 (0.60)
Activities were useful	3.86 (0.94)
Message board was useful	3.64 (1.03)
Logbook was useful	4.23 (0.87)
Navigation links were easy	4.05 (0.72)
I liked the personal login	4.27 (0.63)
I liked being able to communicate with others	3.41 (1.05)
I liked the email counselling	4.14 (0.89)

### Study Objective Two: Preliminary efficacy

#### Intervention

Repeated measures of analyses of covariance (RM ANCOVA) for physical activity behaviour(including baseline physical activity as a covariate), revealed a significant group × time interaction for unweighted moderate and vigorous minutes of physical activity [*F*(1,45) = 4.00, partial-eta squared = 0.08, p = 0.052] (see Table 4), which according to Cohen represents a moderate effect size.[54] However, RM ANCOVA for physical activity behaviour did not reveal a significant group × time interaction for MET.minutes [*F*(1,45) = 1.88, partial-eta squared = 0.04, p < 0.177]. The intervention group participated in more unweighted moderate and vigorous minutes than the control group with a mean difference of 47 minutes (95% CI = -.37-102.66, p < 0.052) (see Table 5). In addition the intervention group participated in more MET.minutes than the control group with a mean difference of 164 MET.minutes (95% CI = -83.12–436.87, p < 0.177). Although not included in our hypotheses, resistance training (including baseline RT as a covariate) was also examined. RM ANCOVA for these two variables did not reveal a significant group × time interaction (see Table 4).

**Table 4:** Physical activity behaviour (n = 47)

	**Control Group n = 24**		Intervention Group n = 23*				
**Variable**	**Time 1** **M (SD)**	**Time 2** **M (SD)**	**Time 1** **M (SD)**	**Time 2** **M (SD)**	**P **(*for interaction*)	**F**	**eta^2^**
MET minutes (per/wk)	501 (582)	490 (562)	483 (620)	654 (659)	0.177	1.88	0.04
adjusted for BMI					**0.043**	4.37	0.09
Unweighted mod & vig (per/wk)	111 (123)	92 (93)	105 (140)	140 (138)	**0.052**	4.00	0.08
adjusted for BMI					**0.010**	7.33	0.15
RT minutes (per/wk)	8 (23)	15 (25)	32 (62)	26 (56)	0.064	3.60	0.07
adjusted for BMI					0.061	3.70	0.08

* Two individuals did not complete baseline Godin measures, therefore, not allowing the last observation to be carried forward technique to be employed. M = mean; SD = standard deviation; Vig = vigorous; Mod = moderate

**Table 5:** Change Scores (n = 47)

**Variable**	**Change score **(between group)	**95% Confidence Interval**		**t**	**p**	**eta^2^**
		Lower Bound	Upper Bound			
MET.minutes (per/wk)	164 (97)	-83.12	436.87	1.37	0.177	0.04
adjusted for BMI	168 (85)	9.66	553.18	2.09	**0.043**	0.09
unweighted mod. & vig mins (per/wk)	47 (45)	-0.370	102.00	2.00	**0.052**	0.10
adjusted for BMI	50 (44)	18.27	125.22	2.71	**0.010**	0.15

Repeated measures of analyses of covariance (RM ANCOVA) for physical activity behaviour (using both baseline physical activity and BMI as covariates), revealed a significant group × time interaction for unweighted moderate and vigorous minutes [*F*(1,44) = 7.33, partial-eta squared = .15, p = 0.010] and MET.minutes [*F*(1,44) = 4.36, partial-eta squared = .09, p < 0.043] of total physical activity, which in both cases indicates a moderate-to-large effect size (see Table 4) [54]. The intervention group participated in more unweighted moderate and vigorous minutes of physical activity than the control group with a mean difference of 50 minutes (95% CI = 18.27 – 125.22, p < 0.010), further, the intervention group participated in more MET.minutes then the control group with a mean difference of 168 MET.minutes (95% CI = 9.66 – 553.18, p < 0.043) (see Table 5). Resistance training (using both baseline resistance training and BMI as covariates) did not reveal a significant group × time interaction effect (see Table 4).

Differences in social cognitive variables (see Table 6), controlling for baseline difference, showed a significant interaction effect for behavioural capacity [*F*(1,44) = 6.12, partial-eta squared = 0.12, p < 0.05]. No other interactions were significant for the remaining social cognitive variables examined (i.e., self-efficacy, outcome expectations, outcome expectancies, self-regulation, situation, reinforcement, social support, emotional coping response, and observational learning).

**Table 6:** Social cognitive variables (n = 49)

	**Control Group** **n = 24**		**Intervention Group** **n = 25**				
**Variable**	**Time 1** **M (SD)**	**Time 2** **M (SD)**	**Time 1** **M (SD)**	**Time 2** **M (SD)**	**p**	**F**	**eta**^2^
Self-efficacy	3.01 (0.71)	2.82 (0.84)	3.00 (0.74)	2.99 (0.84)	0.311	1.05	0.02
Outcome expectations	4.62 (0.37)	4.57 (0.42)	4.46 (0.87)	4.57 (0.54)	0.763	0.92	0.00
Outcome expectancies	2.71 (0.34)	2.65 (0.36)	2.73 (0.29)	2.69 (0.39)	0.799	0.07	0.00
Self-regulation	3.94 (0.53)	3.75 (1.05)	3.81 (0.54)	3.56 (0.91)	0.550	0.36	0.00
Situation	2.11 (0.55)	2.16 (0.59)	2.41 (0.66)	2.35 (0.67)	0.406	0.71	0.02
Reinforcement	2.58 (0.84)	2.70 (0.91)	2.73 (0.66)	2.83 (0.74)	0.961	0.00	0.00
Social support	4.58 (1.99)	4.27 (2.05)	5.06 (1.52)	4.18 (2.10)	0.201	1.69	0.04
Emotional coping resp.	2.78 (0.67)	2.90 (0.67)	2.93 (0.76)	2.90 (0.67)	0.733	0.12	0.00
Behavioural capacity	3.25 (0.98)	2.97 (0.98)	3.10 (1.18)	3.23 (1.32)	**0.001**	7.63	0.14
Observational learning	3.83 (1.32)	3.31 (1.31)	4.14 (0.78)	3.96 (0.92)	0.087	3.05	0.06

The results for objective two were also analysed deleting the 5 cases (2 treatment; 3 controls) who did not complete the post-test. There were no meaningful differences in the results between the deletion and the last observation carried forward techniques. Further, we re-ran the main analyses, adjusting for computer usage (i.e., proficiency of computer use and number of hours/week on the computer), which did not alter the current results.

## Discussion

The first objective of this study was to determine the feasibility (*recruitment, retention, adherence *and *satisfaction*) of the internet in the delivery of physical activity related information to individuals with type 2 diabetes. There is an increasing need to deliver cost-effective physical activity interventions to large numbers of individuals living with this chronic disease. With internet and email becoming a primary mode of communication, an opportunity exists to utilize information technology to elicit physical activity behaviour change.

Recruitment for the Diabetes NetPLAY study did prove to be challenging. Various recruitment methods were employed to attract potential participants including newspaper ads, posters, internet ads and in-person visits to Diabetes Education Centres. Recruitment continued over a four month period with more than 200 study information packages distributed. A large proportion of individuals reported hesitation with using the internet or not having regular access to a computer as reasons for not participating. Although internet usage is continuing to rise, there are still a number of individuals with limited or no internet experience. Lack of interest in physical activity, the 12 week time commitment and the chance of being randomized to the control group were also cited as reasons for not participating in the study. Similar studies have also encountered difficulty in recruiting participants. Napolitano and colleagues [13] had 275 individuals express interest in their internet-based physical activity intervention, but after repeated attempts, only 96 of those participants responded to follow-up. Tate also encountered low recruitment rates in their internet weight loss program in which 196 individuals responded to the study but only 114 followed up with screening. [15]

Results demonstrated that retention of participants to the Diabetes NetPLAY website was high. Forty-four participants completed the post-test questionnaire, representing a 90% retention rate for this study. Similar studies have also demonstrated comparable retention rates. The D-Net intervention by McKay and colleagues [11] revealed a retention rate of 87% at the 8-week follow-up point. Similarly, Napolitano et al. [13] showed retention rates of 88% and 80% at one- and three-month follow-ups, respectively. Finally, 86% of participants completed the six-week pilot intervention conducted by Richardson and colleagues [22]. However, in a recent review by Vandelanotte, Spathonis, Eakin and Owen [8], they reported retention rates to be lower, with an overall average of 73% attrition.

In the review by Vandelanotte and colleagues [8], adherence to internet-based physical activity interventions has been low. Of the five studies that reported objective data, all indicated a decline in website usage as the intervention progressed. In the Diabetes NetPLAY study, over half the intervention group (60%) showed a decline in login frequency while, interestingly, 32% of participants actually increased their usage of the intervention website after the study mid-point. As with non-internet-based studies, adherence continues to be a challenge in the long-term.

The D-Net diabetes self-management program [18], showed a significant decrease in login frequency (50%) during the 10 month intervention. The highest rates of website usage were recorded within the first three months of the program with a gradual drop-off over time. Leslie and colleagues [55] reported on the engagement and retention of participants in the Active Living Online study [56]. Over the 8-week intervention, only 46% of recruited participants visited the website with 77% of those hits recorded in the first 2 weeks of the intervention. The relatively low level of website interactivity may have contributed to the low adherence rates demonstrated in the Active Living Online study, as many participants felt it unnecessary to continue visiting a static website.

In addressing the adherence issue, one might want to consider the interactive features of a physical activity website. Increasing the interactivity of these types of interventions has been suggested in the literature as important components to incorporate into the website design [13,55]. Email, chat rooms, on-line logbooks and updated information may be simple features that aid in the adherence of participants to a physical activity website.

Our Diabetes NetPLAY study used several interactive techniques in an attempt to keep study participants engaged. Individualized emails were sent on a weekly basis, providing general feedback on the specific topic of the week, progress and motivation. An on-line logbook allowed participants to track their progress and receive feedback from their counsellor on how they were doing. The study website was also updated on a weekly basis, with past weeks being archived for future reference. The study message board had various topics posted by the study counsellor at which point, participants could share their thoughts and feelings with others involved in the study. These features may have given participants reason to continue visiting the website on a regular basis throughout the study.

Previous studies have reported a positive correlation between login frequency and behaviour change. Tate, Wing and Winnett [15] found a significant correlation (p < 0.01) between login frequency and weight change between 0 and 6 months time in both the intervention and control groups. While McKay et al. [11] found a significant relation between website usage and greater change in moderate-to-vigorous physical activity within their 8-week intervention. Website usage in this study was monitored by the number of hits to the Diabetes NetPLAY website by each participant. Analysis failed to show a positive association between website usage and physical activity behaviour change. In other words, those who visited the website more frequently didn't report an increase in physical activity behaviour than those who used it less often. Perhaps, there is a threshold regarding the extent to which accessing information yields corresponding behaviour change gains.

Satisfaction among intervention participants for this mode of delivery was positive. Participants indicated they were more satisfied with the personal email counselling than the peer-to-peer support through the message board. A similar finding was also captured by McKay and colleagues [11] with 88% of participants reporting satisfaction with a personal coach versus 35% reported for peer-to-peer support. Additional promotion and encouragement may be needed to generate strong peer-to-peer support systems with this mode of delivery.

Slightly less than half (49%) of study participants reported using the internet on a daily basis while at the same time 43% reported using the internet less than 9 hours in the previous month. This may indicate that while individuals are accessing the internet on a regular basis, they are not spending comparative amounts of time surfing the web. This might be an important aspect to consider when developing further web-based studies as individuals may not be accustomed to spending the required time on study websites to take full advantage of their behaviour change potential.

The second study objective was to determine the preliminary efficacy of the internet as a mode of delivery for eliciting recommended changes in physical activity related cognitions and behaviours for individuals with type 2 diabetes. The results demonstrate that the internet and interactive technology is an efficacious vehicle for promoting physical activity behaviour change among individuals with type 2 diabetes. The intervention group significantly increased their mean total physical activity levels whereas the control group demonstrated a decline in activity levels. Although not all participants in the intervention reached recommended guidelines within the parameters of this study, participants did demonstrate a gradual progression towards the clinical recommendations. The Canadian Diabetes Association [4] recommends individuals with type 2 diabetes gradually increase their activity levels to 150 minutes of moderate intensity, physical activity per week.

The physical activity changes demonstrated in this study have both research and clinical significance. Although our sample size was small, moderate to large effect sizes were demonstrated for physical activity behaviour. Additionally, the weekly average increase of 47 minutes in physical activity behaviour over the course of the study among the intervention group participants has very important practical and clinical implications for public health. The need for cost-effective physical activity interventions that can reach large numbers of people is vital in the public health system. Therefore, these results can also be viewed as meaningful at a population level.

A systematic review of the literature suggests modest effects for the efficacy of web-based physical activity interventions, with just over half of the studies reporting significant positive behavioural changes [8]. Only a few studies have shown effects on physical activity behaviour [13,19,57] while others with [58] or without tailored feedback [15,56,59] have shown no effects on physical activity behaviour. The Diabetes NetPLAY study was able to demonstrate group and time effects with moderate to large effect sizes for physical activity behaviour change, despite having a small sample size.

Plotnikoff et al. [19] found similar results in a workplace context over the same time period. Although effect sizes were small, intervention participants showed an increase in total activity levels at 3 months while activity levels of control participants declined during the same time frame. Likewise, Napolitano and colleagues [13] found that after one month of exposure to an SCT-grounded physical activity website and weekly email tip sheets, intervention participants exhibited higher levels of moderate physical activity and walking minutes compared to the control group. However, at the three-month time-point, only walking minutes remained significant between the two groups.

Similar findings to the Diabetes NetPLAY study were also demonstrated in a study of 434 healthy adults [57]. Researchers found a significant group × time interaction effect in favour of both intervention groups (with or without repeated feedback) for active transportation and leisure-time physical activity compared with the control group. However, in contrast to Diabetes NetPLAY, Vandelanotte et al. [8] were able to show behaviour changes over a longer study period of 6 months.

The Diabetes Network (D-Net) Active Lives Physical Activity Intervention [11] also demonstrated comparable findings to that of Diabetes NetPLAY. Significant time effects in both walking and moderate-to-vigorous intensity physical activity were found at the end of the 8-week study. Unlike NetPLAY, however, McKay and colleagues [11] failed to show a significant difference in physical activity behaviour between intervention and control groups over a shorter period of time. Interestingly, the D-Net study allowed the control group access to diabetes-specific articles in the website library in addition to real-time glucose tracking with graphic feedback for the duration of the 8-week study. Control participants could have been motivated and guided by information accessed through the virtual library and therefore, increased their physical activity behaviour as well.

The significant changes in physical activity behaviour among intervention participants in the Diabetes NetPLAY study may be attributed to several factors. First, individuals may have already been motivated to participate in physical activity prior to study initiation. The simple fact that individuals were interested in participating in a physical activity study may speak to the stage of readiness that many of the participants were in prior to starting the intervention. Second, web-based interventions are still seen as a novel approach for the delivery of health-related physical activity and counselling information. Therefore, participants could have been motivated to increase their physical activity behaviour, in part, because of the novel mode of delivery and not the information, per se. This speaks to the importance of further research in this area.

The intervention group in our study also demonstrated an increase in behavioural capacity (i.e., self-reported ability to complete various physical tasks) over the course of the 12-week study. Participants in the intervention group felt they were better able to complete physical tasks like walking and jogging after taking part in Diabetes NetPLAY. However, there were no significant increases in any other physical activity related cognitions. These findings contrast similar studies grounded in theoretical concepts. Plotnikoff and colleagues [19] measured several physical activity-related cognitions from a variety of social cognitive theories. Following the 12-week study, participants in the intervention group were more efficacious in four out of the seven social-cognitive variables measured, including self-efficacy.

The fact the majority of physical activity-related cognitions did not significantly change in this study may be explained by the *response shift theory *concept. Borrowed from the quality of life domain, this concept proposes that an individual's self-perception and/or internal standards (e.g., self-regulation, outcome expectations) shift as the result of a change in a measurable behaviour (e.g., physical activity) [60]. In other words, as an individuals behaviour changes (i.e., they become more active), they encounter new situations, barriers etc. as a result, causing a shift in their cognitions related to that particular behaviour (i.e., physical activity). Examination of the response shift theory has occurred primarily in the quality of life domain; therefore, additional research in the social cognitive field is needed to evaluate its true effectiveness in the physical activity domain.

A second explanation for the lack of change in behaviour-related cognitions could be attributed to elevated self-report measures at baseline. This could have produced a ceiling effect, leaving limited room for improvement in these social cognitive variables.

Strengths of this study include, first, the comprehensive use of a theoretically-based framework in the messaging and composition of the website tools. The use of theoretically-based interventions may strengthen program outcomes and facilitate successful behaviour change [61,62]. Second, the use of SCT in its entirety for the design of a web-based program is a unique feature as compared to a large portion of the programs which tend to operationalize only specific components of social cognitive theories. Finally, this study contributes to the growing body of literature of web-based mediums for the delivery of health information. The use of interactive features such as linked email counselling, message boards, and logbooks are important tools in the further development of knowledge in this area.

This study, however, is not without its limitations. Although we were able to detect physical activity behaviour differences, the limited sample size may have prevented the detection of differences between many social cognitive variables, as this pilot study was not sufficiently powered [54]. The lack of objective physical activity measures and the dependence on self-report indicators could be considered a limitation [63]. The use of objective physical activity and health status measures, such as accelerometers and blood tests respectively, would be important for future studies on this topic. Although we did see an increase in physical activity behaviour among the intervention participants, we were not able to tease out the effects of the SCT messaging versus other components of the website. Therefore, it is difficult to pinpoint which components/messages had the most, if any, effect. Finally, there was no long-term follow-up which prevented an evaluation of the intervention's long-term efficacy.

Future research should explore the use of web-based interventions to sustain long-term behaviour change within a broad range of demographic categories. Researchers should attempt to recruit and engage larger samples of individuals with type 2 diabetes over the longer-term. Additionally, future studies may benefit from the inclusion of various follow-up assessments including process evaluations (e.g., focus groups, interviews) and detailed statistics (e.g., frequency and duration of access) related to patterns of website use. Results of such feedback could further inform the utility and applicability of such websites. Further exploration in the grounding of messages and information in specific health behaviour change theories and models is an important step likely to improve the success of such interventions. The use of other social cognitive theories and models as frameworks for further web-based interventions should also be considered in future research. Further, multi-level, ecological (i.e., social, organizational, community, policy, and built environmental) strategies should also be considered for implementation with individual-based, web-based approaches to elicit physical activity behaviour change in this population.

Tailored messaging and interactive website features should be examined to further determine if such elements can maintain long-term engagement and adherence to programming. Increasing the usage of various internet items (i.e., message board, logbook) should be explored in further detail to examine their effects on successful behaviour change in this population.

Examining the use of algorithms in the design of web-based physical activity interventions would also be of value. Allowing participants to receive instantaneous, tailored feedback may increase adherence and physical activity behaviour change. In addition, the use of graphs or charts to provide visual feedback on behaviour change may appeal to and further motivate participants. Future studies should track website usage through a variety of methods including length of time participants spend viewing website pages and their specific use of the website applications.

Finally, the use of the internet to deliver behaviour-change interventions could present additional barriers for some individuals. Therefore, it would be beneficial for researchers to determine how to better relate with participants and understand the barriers-to-use so as to better appeal to more individuals.

In summary, this study revealed the web-based delivery of physical activity programs holds particular promise for behaviour change in the diabetes population. The expanding availability of the internet allows such programs to reach large numbers of individuals while at the same time providing instantaneous support and feedback. Although in its relative infancy, web-based physical activity interventions, such as NetPLAY, are important in the development and expansion of future research in this area.

## Competing interests

The authors declare that they have no competing interests.

## Authors' contributions

TL and RCP conceived the study. RCP, TL, KSC and NB were involved in the survey design. TL coordinated survey delivery and data collection. TL analyzed the data. TL, RCP, KSC and NB interpreted the data. TL and RCP were responsible for drafting the manuscript. All authors critically evaluated the article for content and approved the final version.
